# Using transfer entropy to study synaptic integration in Purkinje cells

**DOI:** 10.1186/1471-2202-16-S1-P236

**Published:** 2015-12-18

**Authors:** Kirsty Kidd, Neil Davey, Daniel Polani, James M Bower, Volker Steuber

**Affiliations:** 1 School of Computer Science, University of Hertfordshire, Hatfield, UK

## 

Information theory has been used in many ways to analyse the output of neurons and how it relates to the input. Mutual information is often used as a means to describe this relationship [1], but as it is symmetrical, it does not necessarily describe how much information is transferred specifically from input to output. An alternative method, transfer entropy [2], introduces directionality to the analysis of this problem. Transfer entropy allows one to study how the predictability of the output of the cell is affected by varying time windows of preceding input spike trains.

In this work we are using two different extensions to transfer entropy that attempt to limit bias. The first method of estimating transfer entropy we are using is as described by Wibral et al [3], which places a restriction of a single time step on the length of the delay considered on the destination time series. This restriction is placed to avoid overestimating the information transferred from input to output by ensuring that the information provided by the history of the output has not been underestimated. The second approach, put forward by Gourévitch and Eggermont [4] creates a 'shuffled' transfer entropy value from the average transfer entropy, taken over many runs where the history of the input is randomly shuffled. The shuffled value can then be used to normalise the transfer entropy of the un-shuffled data.

We are currently investigating the use of transfer entropy to study the output of an active Purkinje cell model [5,6] in response to a gamma-distributed input, while varying the location of the input site. Active channels in dendrites allow the effect of input to be location-independent, but using transfer entropy enables us to highlight differences in delay of information transfer based on the morphology of the cell.
